# Di-μ_2_-chlorido-dichloridoocta­methyldi-μ_3_-oxido-tetra­tin(IV) bis[chloridodimeth­yl(pyrrolidine-1-carbodithio­ato-κ^2^ *S*,*S*′)tin(IV)]

**DOI:** 10.1107/S1600536812017485

**Published:** 2012-04-25

**Authors:** Nicolás Rodríguez, Pedro F. B. Brandão, Coco K. Y. A. Okio

**Affiliations:** a Departamento de Química, Universidad Nacional de Colombia, Sede Bogotá, Bogotá, Colombia

## Abstract

In the title co-crystal, [Sn_4_(CH_3_)_8_Cl_4_O_2_]·2[Sn(CH_3_)_2_Cl(C_4_H_8_NS_2_)], all the Sn^IV^ atoms are in distorted trigonal–bipyramidal environments. In the mononuclear species, the carbodithio­ate ligand is unsymmetrically coordinated to the Sn^IV^ atom, with Sn—S distances of 2.6722 (12) and 2.4706 (11) Å. All atoms with the exception of the methyl groups and one of the pyrrolidine ring CH_2_ groups lie on a crystallographic mirror plane. The pyrrolidine ring exhibits an envelope conformation; the C atom at the flap is disordered above and below the plane of symmetry with fixed occupation factors of 0.50. The centrosymmetric dimer species consists of a central Sn_2_O_2_ unit with two adjacent Sn_2_OCl four-membered rings.

## Related literature

For related structures, see: Graziani *et al.* (1983 ▶); Othman *et al.* (1997 ▶); Cortes *et al.* (2010 ▶). For biological applications of organotin(IV) complexes, see: Davies & Smith (1982 ▶).
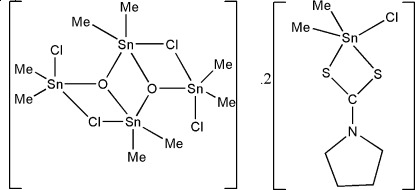


## Experimental

### 

#### Crystal data

[Sn_4_(CH_3_)_8_Cl_4_O_2_]·2[Sn(CH_3_)_2_Cl(C_4_H_8_NS_2_)]
*M* *_r_* = 1429.74
Orthorhombic, 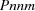

*a* = 14.5262 (4) Å
*b* = 14.6086 (6) Å
*c* = 10.8338 (4) Å
*V* = 2299.01 (14) Å^3^
*Z* = 2
Mo *K*α radiation
μ = 3.76 mm^−1^
*T* = 193 K
0.18 × 0.09 × 0.06 mm

#### Data collection

Nonius KappaCCD diffractometer
Absorption correction: multi-scan (*SORTAV*; Blessing, 1995 ▶) *T*_min_ = 0.640, *T*_max_ = 0.749
11647 measured reflections
2768 independent reflections
2450 reflections with *I* > 2σ(*I*)
*R*_int_ = 0.039

#### Refinement

*R*[*F* ^2^ > 2σ(*F* ^2^)] = 0.028
*wR*(*F* ^2^) = 0.066
*S* = 1.12
2767 reflections
124 parameters
H-atom parameters constrained
Δρ_max_ = 0.75 e Å^−3^
Δρ_min_ = −1.07 e Å^−3^

### 

Data collection: *COLLECT* (Nonius, 1998 ▶); cell refinement: *DENZO* (Otwinowski & Minor, 1997 ▶); data reduction: *DENZO*; program(s) used to solve structure: *SHELXS97* (Sheldrick, 2008 ▶); program(s) used to refine structure: *SHELXL97* (Sheldrick, 2008 ▶); molecular graphics: *PLATON* (Spek, 2009 ▶); software used to prepare material for publication: *SHELXL97*.

## Supplementary Material

Crystal structure: contains datablock(s) I, global. DOI: 10.1107/S1600536812017485/lr2051sup1.cif

Structure factors: contains datablock(s) I. DOI: 10.1107/S1600536812017485/lr2051Isup2.hkl

Additional supplementary materials:  crystallographic information; 3D view; checkCIF report

**Fig. 1.**
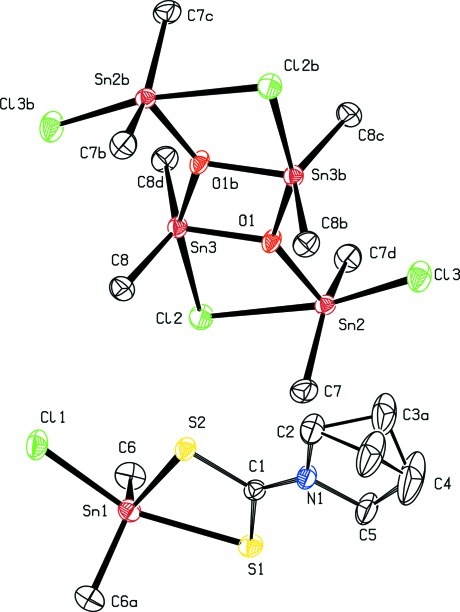
The molecular structure of (I). Displacement ellipsoids are drawn at the 40% probability level. H atoms have been omitted.

Enhanced figure: interactive version of Fig. 1

## supplementary crystallographic information

### Comment

Organotin(IV) complexes have been extensively studied due to the diversity of structures that such compounds can form and to their potential biological activities as well as their wide industrial and agricultural applications (Davies & Smith, 1982). In the framework of our research for new organotin(IV) compounds (Cortes *et al.*, 2010), we report here the obtention of the title compound. In an attempt to study the biological applications of complexes prepared from dimethyltin dichloride and pyrrolidinedithiocarbamate, the title compound has been obtained. Separately, both crystals have been reported (Graziani *et al.* 1983) and (Othman *et al.* 1997). The molecular structure and the atom-numbering scheme of the title compound are shown in Fig.1. It's a mononuclear/tetranuclear Sn^IV^ cocrystal. In the mononuclear diorganotin dithiocarbamate species, the tin atom is five-coordinated, being chelated by an asymmetrically coordinating dithiocarbamate ligand, a chloride and two methyl groups. The Sn—S bond distance (2.6708 (15) Å) aproximately *trans*- to the chloride atom is longer than the other Sn—S bond distance (2.4714 (14) Å) as reported by Othman *et al.* (1997). The centrosymmetric dimeric species bears a central part which consists of Sn_2_O_2_ ring with two adjacent Sn_2_OCl four-membered rings. The Sn, O and Cl atoms are nearly coplanar and the Sn (IV) atoms are in a distorted trigonal bipyramidal geometry. This behaviour is also consistent with the reported structure (Graziani *et al.* 1983).

### Experimental

Compound (I) was obtained by reacting dimethyltin (IV) dichloride with sodium pyrrolidinecarbodithioate in methanol under reflux for 3 h. Colourless crystals suitable for X-ray analysis were grown by slow solvent evaporation.

### Refinement

H atoms were positioned geometrically, with C—H distances of 0.96 to 1.00 Å, and constrained to ride on their parent atoms, with *U*iso(H) = 1.2–1.5U_eq_(C).

### Crystal data

**Table:**

[Sn_4_(CH_3_)_8_Cl_4_O_2_]·2[Sn(CH_3_)_2_Cl(C_4_H_8_NS_2_)]	*F*(000) = 1360
*M**_r_* = 1429.74	*D*_x_ = 2.065 Mg m^−^^3^
Orthorhombic, *P**n**n**m*	Mo *K*α radiation, λ = 0.71073 Å
Hall symbol: -P 2 2n	Cell parameters from 11648 reflections
*a* = 14.5262 (4) Å	θ = 2.0–27.5°
*b* = 14.6086 (6) Å	µ = 3.76 mm^−^^1^
*c* = 10.8338 (4) Å	*T* = 193 K
*V* = 2299.01 (14) Å^3^	Prism, colorless
*Z* = 2	0.18 × 0.09 × 0.06 mm

### Data collection

**Table:**

Nonius KappaCCD diffractometer	2768 independent reflections
Radiation source: fine-focus sealed tube	2450 reflections with *I* > 2σ(*I*)
Graphite monochromator	*R*_int_ = 0.039
π scans	θ_max_ = 27.5°, θ_min_ = 2.7°
Absorption correction: multi-scan (*SORTAV*; Blessing, 1995)	*h* = −18→14
*T*_min_ = 0.640, *T*_max_ = 0.749	*k* = −18→15
11647 measured reflections	*l* = −14→12

### Refinement

**Table:**

Refinement on *F*^2^	Primary atom site location: structure-invariant direct methods
Least-squares matrix: full	Secondary atom site location: difference Fourier map
*R*[*F*^2^ > 2σ(*F*^2^)] = 0.028	Hydrogen site location: inferred from neighbouring sites
*wR*(*F*^2^) = 0.066	H-atom parameters constrained
*S* = 1.12	*w* = 1/[σ^2^(*F*_o_^2^) + (0.0315*P*)^2^ + 1.1007*P*] where *P* = (*F*_o_^2^ + 2*F*_c_^2^)/3
2767 reflections	(Δ/σ)_max_ = 0.002
124 parameters	Δρ_max_ = 0.75 e Å^−^^3^
0 restraints	Δρ_min_ = −1.07 e Å^−^^3^

### Special details

**Table:**

Experimental. Absorption correction: multi-scan from symmetry-related measurements (SORTAV; Blessing, 1995)
Geometry. All esds (except the esd in the dihedral angle between two l.s. planes) are estimated using the full covariance matrix. The cell esds are taken into account individually in the estimation of esds in distances, angles and torsion angles; correlations between esds in cell parameters are only used when they are defined by crystal symmetry. An approximate (isotropic) treatment of cell esds is used for estimating esds involving l.s. planes.
Refinement. Refinement of *F*^2^ against ALL reflections. The weighted *R*-factor *wR* and goodness of fit *S* are based on *F*^2^, conventional *R*-factors *R* are based on *F*, with *F* set to zero for negative *F*^2^. The threshold expression of *F*^2^ > σ(*F*^2^) is used only for calculating *R*-factors(gt) *etc*. and is not relevant to the choice of reflections for refinement. *R*-factors based on *F*^2^ are statistically about twice as large as those based on *F*, and *R*- factors based on ALL data will be even larger.

### Fractional atomic coordinates and isotropic or equivalent isotropic displacement parameters (Å^2^)

**Table:**

	*x*	*y*	*z*	*U*_iso_*/*U*_eq_	Occ. (<1)
Sn2	0.315166 (18)	0.632255 (19)	0.0000	0.02461 (9)	
Sn3	0.559454 (18)	0.59668 (2)	0.0000	0.02538 (9)	
Cl2	0.47009 (7)	0.75444 (7)	0.0000	0.0361 (3)	
Cl3	0.21017 (8)	0.50149 (8)	0.0000	0.0478 (3)	
O1	0.42728 (18)	0.5486 (2)	0.0000	0.0295 (7)	
C7	0.3016 (2)	0.6704 (3)	0.1864 (3)	0.0430 (9)	
H7A	0.3520	0.6434	0.2344	0.064*	
H7B	0.2425	0.6483	0.2183	0.064*	
H7C	0.3041	0.7372	0.1933	0.064*	
C8	0.6065 (2)	0.6172 (2)	0.1817 (3)	0.0382 (8)	
H8A	0.5582	0.5995	0.2401	0.057*	
H8B	0.6219	0.6819	0.1935	0.057*	
H8C	0.6614	0.5796	0.1961	0.057*	
Sn1	0.02288 (2)	0.29537 (2)	0.0000	0.02941 (10)	
Cl1	−0.14100 (8)	0.23939 (8)	0.0000	0.0442 (3)	
S1	0.20483 (8)	0.26837 (8)	0.0000	0.0322 (3)	
S2	0.05638 (7)	0.12957 (7)	0.0000	0.0295 (3)	
N1	0.2337 (2)	0.0884 (2)	0.0000	0.0309 (9)	
C1	0.1741 (3)	0.1552 (3)	0.0000	0.0258 (9)	
C2	0.2102 (3)	−0.0107 (3)	0.0000	0.0472 (14)	
H2A	0.1704	−0.0212	−0.0698	0.057*	0.50
H2B	0.1704	−0.0212	0.0698	0.057*	0.50
C3	0.2990 (5)	−0.0522 (6)	0.0561 (11)	0.088 (5)	0.50
H3A	0.2989	−0.0477	0.1473	0.106*	0.50
H3B	0.3069	−0.1170	0.0315	0.106*	0.50
C4	0.3721 (5)	0.0077 (5)	0.0000	0.073 (5)	
H4A	0.4096	−0.0081	0.0740	0.151*	0.50
H4B	0.4096	−0.0081	−0.0740	0.151*	0.50
C5	0.3333 (3)	0.1031 (4)	0.0000	0.0505 (15)	
H5A	0.3529	0.1373	0.0744	0.061*	0.50
H5B	0.3529	0.1373	−0.0744	0.061*	0.50
C6	0.0147 (3)	0.3637 (2)	−0.1722 (3)	0.0443 (9)	
H6A	−0.0440	0.3966	−0.1777	0.067*	
H6B	0.0187	0.3188	−0.2392	0.067*	
H6C	0.0657	0.4074	−0.1792	0.067*	

### Atomic displacement parameters (Å^2^)

**Table:**

	*U*^11^	*U*^22^	*U*^33^	*U*^12^	*U*^13^	*U*^23^
Sn2	0.02385 (15)	0.02385 (16)	0.02612 (18)	0.00354 (11)	0.000	0.000
Sn3	0.02293 (16)	0.02345 (16)	0.02978 (19)	−0.00087 (11)	0.000	0.000
Cl2	0.0334 (5)	0.0236 (5)	0.0513 (8)	0.0011 (5)	0.000	0.000
Cl3	0.0336 (6)	0.0302 (6)	0.0796 (10)	−0.0038 (5)	0.000	0.000
O1	0.0185 (13)	0.0209 (14)	0.049 (2)	0.0031 (12)	0.000	0.000
C7	0.0401 (18)	0.062 (2)	0.027 (2)	0.0047 (17)	0.0042 (15)	−0.0051 (18)
C8	0.0393 (18)	0.0421 (19)	0.033 (2)	0.0004 (15)	−0.0041 (15)	−0.0050 (16)
Sn1	0.03238 (17)	0.02334 (16)	0.0325 (2)	0.00254 (12)	0.000	0.000
Cl1	0.0275 (5)	0.0356 (6)	0.0694 (9)	0.0064 (5)	0.000	0.000
S1	0.0309 (5)	0.0253 (5)	0.0405 (7)	−0.0071 (4)	0.000	0.000
S2	0.0227 (5)	0.0235 (5)	0.0421 (7)	−0.0021 (4)	0.000	0.000
N1	0.0206 (17)	0.030 (2)	0.042 (2)	−0.0001 (15)	0.000	0.000
C1	0.026 (2)	0.027 (2)	0.024 (2)	−0.0029 (18)	0.000	0.000
C2	0.032 (2)	0.025 (2)	0.085 (4)	0.000 (2)	0.000	0.000
C3	0.039 (4)	0.042 (4)	0.184 (16)	0.008 (4)	−0.011 (5)	0.018 (6)
C4	0.038 (3)	0.060 (5)	0.120 (15)	0.014 (3)	0.000	0.000
C5	0.024 (2)	0.041 (3)	0.087 (5)	−0.002 (2)	0.000	0.000
C6	0.056 (2)	0.0368 (19)	0.040 (2)	0.0009 (17)	−0.0034 (18)	0.0055 (16)

### Geometric parameters (Å, º)

**Table:**

Sn2—O1	2.036 (3)	S1—C1	1.712 (4)
Sn2—C7	2.104 (4)	S2—C1	1.751 (4)
Sn2—C7^i^	2.104 (4)	N1—C1	1.305 (6)
Sn2—Cl3	2.4445 (12)	N1—C5	1.462 (6)
Sn2—Cl2	2.8724 (11)	N1—C2	1.488 (6)
Sn3—O1	2.044 (3)	C2—C3	1.550 (9)
Sn3—C8	2.105 (3)	C2—C3^i^	1.550 (9)
Sn3—C8^i^	2.105 (3)	C2—H2A	0.9644
Sn3—O1^ii^	2.132 (3)	C2—H2B	0.9644
Sn3—Cl2	2.6451 (11)	C3—C3^i^	1.22 (2)
O1—Sn3^ii^	2.132 (3)	C3—C4	1.505 (10)
C7—H7A	0.9800	C3—H3A	0.9902
C7—H7B	0.9800	C3—H3B	0.9901
C7—H7C	0.9800	C4—C5	1.504 (8)
C8—H8A	0.9800	C4—C3^i^	1.505 (10)
C8—H8B	0.9800	C4—H4A	0.9959
C8—H8C	0.9800	C4—H4B	0.9959
Sn1—C6^i^	2.119 (4)	C5—H5A	0.9900
Sn1—C6	2.119 (4)	C5—H5B	0.9900
Sn1—S2	2.4706 (11)	C6—H6A	0.9800
Sn1—Cl1	2.5173 (12)	C6—H6B	0.9800
Sn1—S1	2.6722 (12)	C6—H6C	0.9800
			
O1—Sn2—C7	103.51 (10)	C1—N1—C5	123.2 (4)
O1—Sn2—C7^i^	103.51 (10)	C1—N1—C2	125.1 (4)
C7—Sn2—C7^i^	147.4 (2)	C5—N1—C2	111.7 (4)
O1—Sn2—Cl3	91.74 (8)	N1—C1—S1	123.3 (3)
C7—Sn2—Cl3	98.55 (11)	N1—C1—S2	119.2 (3)
C7^i^—Sn2—Cl3	98.55 (11)	S1—C1—S2	117.5 (2)
O1—Sn2—Cl2	75.29 (8)	N1—C2—C3	100.9 (4)
C7—Sn2—Cl2	84.76 (10)	N1—C2—C3^i^	100.9 (4)
C7^i^—Sn2—Cl2	84.76 (10)	C3—C2—C3^i^	46.2 (9)
Cl3—Sn2—Cl2	167.02 (4)	N1—C2—H2A	107.1
O1—Sn3—C8	110.72 (10)	C3—C2—H2A	138.1
O1—Sn3—C8^i^	110.72 (10)	C3^i^—C2—H2A	97.4
C8—Sn3—C8^i^	138.5 (2)	N1—C2—H2B	107.1
O1—Sn3—O1^ii^	75.11 (12)	C3—C2—H2B	97.4
C8—Sn3—O1^ii^	96.44 (10)	C3^i^—C2—H2B	138.1
C8^i^—Sn3—O1^ii^	96.44 (10)	H2A—C2—H2B	103.3
O1—Sn3—Cl2	80.68 (8)	C3^i^—C3—C4	66.2 (5)
C8—Sn3—Cl2	92.04 (10)	C3^i^—C3—C2	66.9 (4)
C8^i^—Sn3—Cl2	92.04 (10)	C4—C3—C2	101.6 (6)
O1^ii^—Sn3—Cl2	155.80 (8)	C3^i^—C3—H3A	176.2
Sn3—Cl2—Sn2	80.97 (3)	C4—C3—H3A	111.4
Sn2—O1—Sn3	123.06 (14)	C2—C3—H3A	111.4
Sn2—O1—Sn3^ii^	132.06 (14)	C3^i^—C3—H3B	74.4
Sn3—O1—Sn3^ii^	104.89 (12)	C4—C3—H3B	111.4
Sn2—C7—H7A	109.5	C2—C3—H3B	111.4
Sn2—C7—H7B	109.5	H3A—C3—H3B	109.4
H7A—C7—H7B	109.5	C5—C4—C3^i^	105.9 (5)
Sn2—C7—H7C	109.5	C5—C4—C3	105.9 (5)
H7A—C7—H7C	109.5	C3^i^—C4—C3	47.7 (9)
H7B—C7—H7C	109.5	C5—C4—H4A	114.8
Sn3—C8—H8A	109.5	C3^i^—C4—H4A	125.2
Sn3—C8—H8B	109.5	C3—C4—H4A	85.8
H8A—C8—H8B	109.5	C5—C4—H4B	114.8
Sn3—C8—H8C	109.5	C3^i^—C4—H4B	85.8
H8A—C8—H8C	109.5	C3—C4—H4B	125.2
H8B—C8—H8C	109.5	H4A—C4—H4B	107.2
C6^i^—Sn1—C6	123.3 (2)	N1—C5—C4	103.6 (4)
C6^i^—Sn1—S2	118.23 (10)	N1—C5—H5A	111.0
C6—Sn1—S2	118.23 (10)	C4—C5—H5A	111.0
C6^i^—Sn1—Cl1	95.75 (11)	N1—C5—H5B	111.0
C6—Sn1—Cl1	95.75 (11)	C4—C5—H5B	111.0
S2—Sn1—Cl1	82.40 (4)	H5A—C5—H5B	109.0
C6^i^—Sn1—S1	97.18 (11)	Sn1—C6—H6A	109.5
C6—Sn1—S1	97.18 (11)	Sn1—C6—H6B	109.5
S2—Sn1—S1	70.16 (3)	H6A—C6—H6B	109.5
Cl1—Sn1—S1	152.55 (4)	Sn1—C6—H6C	109.5
C1—S1—Sn1	83.39 (14)	H6A—C6—H6C	109.5
C1—S2—Sn1	88.99 (15)	H6B—C6—H6C	109.5
			
O1—Sn3—Cl2—Sn2	0.0	Cl1—Sn1—S1—C1	0.0
C8—Sn3—Cl2—Sn2	−110.67 (10)	C6^i^—Sn1—S2—C1	−87.53 (12)
C8^i^—Sn3—Cl2—Sn2	110.67 (10)	C6—Sn1—S2—C1	87.53 (12)
O1^ii^—Sn3—Cl2—Sn2	0.0	Cl1—Sn1—S2—C1	180.0
O1—Sn2—Cl2—Sn3	0.0	S1—Sn1—S2—C1	0.0
C7—Sn2—Cl2—Sn3	105.46 (11)	C5—N1—C1—S1	0.0
C7^i^—Sn2—Cl2—Sn3	−105.46 (11)	C2—N1—C1—S1	180.0
Cl3—Sn2—Cl2—Sn3	0.0	C5—N1—C1—S2	180.0
C7—Sn2—O1—Sn3	−80.78 (11)	C2—N1—C1—S2	0.0
C7^i^—Sn2—O1—Sn3	80.78 (11)	Sn1—S1—C1—N1	180.0
Cl3—Sn2—O1—Sn3	180.0	Sn1—S1—C1—S2	0.0
Cl2—Sn2—O1—Sn3	0.0	Sn1—S2—C1—N1	180.0
C7—Sn2—O1—Sn3^ii^	99.22 (11)	Sn1—S2—C1—S1	0.0
C7^i^—Sn2—O1—Sn3^ii^	−99.22 (11)	C1—N1—C2—C3	−156.4 (5)
Cl3—Sn2—O1—Sn3^ii^	0.0	C5—N1—C2—C3	23.6 (5)
Cl2—Sn2—O1—Sn3^ii^	180.0	C1—N1—C2—C3^i^	156.4 (5)
C8—Sn3—O1—Sn2	88.65 (11)	C5—N1—C2—C3^i^	−23.6 (5)
C8^i^—Sn3—O1—Sn2	−88.65 (11)	N1—C2—C3—C3^i^	−94.7 (2)
O1^ii^—Sn3—O1—Sn2	180.0	N1—C2—C3—C4	−37.2 (6)
Cl2—Sn3—O1—Sn2	0.0	C3^i^—C2—C3—C4	57.6 (6)
C8—Sn3—O1—Sn3^ii^	−91.35 (11)	C3^i^—C3—C4—C5	97.2 (3)
C8^i^—Sn3—O1—Sn3^ii^	91.35 (11)	C2—C3—C4—C5	39.2 (7)
O1^ii^—Sn3—O1—Sn3^ii^	0.0	C2—C3—C4—C3^i^	−58.1 (6)
Cl2—Sn3—O1—Sn3^ii^	180.0	C1—N1—C5—C4	180.0
C6^i^—Sn1—S1—C1	117.48 (10)	C2—N1—C5—C4	0.0
C6—Sn1—S1—C1	−117.48 (10)	C3^i^—C4—C5—N1	24.8 (5)
S2—Sn1—S1—C1	0.0	C3—C4—C5—N1	−24.8 (5)

Symmetry codes: (i) *x*, *y*, −*z*; (ii) −*x*+1, −*y*+1, −*z*.
